# Anaesthesia and Caries Treatment by Dental Hygienists: A Worldwide Review

**DOI:** 10.1016/j.identj.2022.08.013

**Published:** 2022-10-17

**Authors:** Meryam Bozia, Erwin Berkhout, Fridus van der Weijden, Dagmar Else Slot

**Affiliations:** a Department of Oral Radiology, Academic Centre for Dentistry Amsterdam (ACTA), Amsterdam, The Netherlands; b Department of Periodontology, Academic Centre for Dentistry Amsterdam (ACTA), Amsterdam, The Netherlands

**Keywords:** Dental hygienist, Questionnaire, Task redistribution, Local anaesthesia, Caries treatment

## Abstract

**Objectives:**

This study aimed to summarise the competencies and legal position of the dental hygienist (DH) regarding local anaesthesia and caries treatment through a worldwide review.

**Methods:**

A structured and peer-reviewed online questionnaire consisting of 27 questions was developed and emailed to all DH associations that are members of the International Federation of Dental Hygienists or European Dental Hygienists Federation. After obtaining the data, all responding associations were contacted to confirm that the data were summarised in the correct order and were asked to provide further clarification of answers if necessary. A descriptive analysis was performed to summarise the data.

**Results:**

Thirty-one countries were approached and 26 responded, resulting in a response rate of 84%. In 62% of the countries, the DH can administer local anaesthesia via infiltration and/or block anaesthesia. In 23% of the countries, the DH can indicate the placement of a caries restoration. In 15% of the countries, the DH can place caries restorations. In 81% of the countries, the DH can apply sealants.

**Conclusions:**

Considerable variation exists amongst countries regarding the extended scope of DH practice. Overall, independently administering local anaesthesia appears to be more accepted as being within the scope of DH practice than caries removal and the placement of restorations.

## Introduction

Dental hygiene education has evolved and expanded significantly since 1945, surpassing the requirements for a 2-year associate degree.^1^ Dental hygienists (DHs) are trained to assess risk and educate and help patients manage and reduce the risk of oral diseases. There appears to be an ongoing need to involve the DHs in the active management of caries.^2^ For instance, in the Netherlands, the DH is allowed to remove caries and place restorations.^3^, ^4^, ^5^ Worldwide, DHs lobby for expanding the scope of practice to administer local anaesthesia,^6^ particularly because the employment of dentists is not expected to keep pace with the increased demand for dental services.^7^ Expanding the DH's scope of practice with tasks could help to reduce oral health disparities, but the extent to which this is implemented is unexplored.^8^

From a regulatory perspective, the European Dental Hygiene Federation (EDHF) has found it difficult to determine exactly what constitutes the role of a DH.^9^ Similarly, throughout the Asian region, the scope of DH practice is considered unclear.^10^^,^^11^ The need for research and knowledge of the DH profession in terms of the extended scope of practice and education programmes is rising.^9^^,^^12^ The full understanding of the DH role and scope of practice is limited between different countries and results in ineffective and inefficient interprofessional collaboration.^3^^,^^13^

Important research in this field was conducted more than a decade ago in an international longitudinal study.^14^ Since then, more countries have joined the International Federation of Dental Hygiene (IFDH). A recent publication has described the worldwide extended scope of DH practice, focusing on radiology.^15^ Following this publication, the present paper aims to summarise the scope of practice of the DH with a focus on local anaesthesia and caries treatment through a worldwide review.

## Materials and methods

### Study outline, guidelines, and ethics

This paper is part of the project “Worldwide Dental Hygienists Extended Scope of Practice.” The study protocol was approved by the Institutional Review and Ethics Board of the Academic Centre for Dentistry Amsterdam (ACTA; reference code 201913). The manuscript was prepared according to the guidelines of the Strengthening the Reporting of Observational Studies in Epidemiology^16^ and Checklist for Reporting Results of Internet E-Surveys.^17^ The material and methods of this study are identical to the first paper in which the results for the radiology questions are reported,^15^ with the aim to review the competencies and legal position of the DH profession from a global perspective.

### Target group

All national dental hygiene associations that were members of the IFDH or EDHF in 2018 were contacted as the target group. See Table 1 for an overview of the associations from the 31 countries approached.

**Table 1:** Respondents: IFDH and EFDH member countries.

	IFDH Member	EDFH Member	Responded to Questionnaire	Responded to Validation
**Australia**	*√*	*NA*	*√*	*√*
**Austria**	*√*	*√*	*√*	*√*
**Canada**	*√*	*NA*	*√*	*√*
**Czech Republic**	*√*	*√*	*√*	*√*
**Denmark**	*√*	*√*	*√*	*√*
**Finland**	*√*	*√*	*√*	*√*
**Germany**	*√*	*√*	*-*	*NA*
**Ireland**	*√*	*√*	*√*	*√*
**Israel**	*√*	*√*	*√*	*√*
**Italy**	*√*	*√*	*√*	*√*
**Japan**	*√*	*NA*	*√*	*√*
**Korea**	*√*	*NA*	*-*	*NA*
**Latvia**	*√*	*NA*	*√*	*√*
**Lithuania**	*-*	*√*	*√*	*-*
**Malta**	*√*	*√*	*√*	*√*
**Netherlands**	*√*	*√*	*√*	*√*
**Nepal**	*√*	*NA*	*-*	*NA*
**New Zealand**	*√*	*NA*	*-*	*NA*
**Norway**	*√*	*√*	*√*	*√*
**Poland**	*-*	*√*	*√*	*-*
**Portugal**	*√*	*√*	*√*	*√*
**Russia**	*√*	*√*	*√*	*√*
**Singapore**	*√*	*NA*	*√*	*-*
**Slovak Republic**	*√*	*√*	*√*	*√*
**South Africa**	*√*	*NA*	*√*	*-*
**Spain**	*√*	*√*	*√*	*-*
**Sweden**	*√*	*√*	*√*	*√*
**Switzerland**	*√*	*√*	*√*	*√*
**United Arab Emirates**	*√*	*NA*	*-*	*NA*
**United Kingdom**	*√*	*√*	*√*	*-*
**United States of America**	*√*	*NA*	*√*	*√*
**N**	*29*	*20*	*26*	*20*

IFDH, International Federation of Dental Hygiene; EFDH, European Dental Hygiene Federation; NA, not applicable; √, yes; -, no.

Adapted from Bozia et al (2022).

### Questionnaire development

A questionnaire was developed to gather data specifically on the administration of local anaesthesia and caries treatment. To assess the suitability of the questionnaire format, the research team conducted a scoping exercise and pilot study for which the preliminary questionnaire was completed and reviewed by 5 DHs from different countries. It was also peer-reviewed by the IFDH, EDHF, and the Dutch Dental Hygienist Association (Nederlandse Vereniging van Mondhygiënisten, NVM) to ensure its comprehensibility and usability.

This pilot study aided in rephrasing some of the questions with commonly used and undisputed English terminology. The 27-item online questionnaire included general questions regarding education, independent practice, task-delegating authority to auxiliary personnel, and indirect access to patients for dental hygiene care. The questionnaire included questions related to the legal status of tasks in cariology and administering local anaesthesia. The closed-ended questions in this questionnaire could potentially limit the richness of the potential answers. This issue was overcome by adding an open-ended question at the end of each category. Open- and closed-ended questions allowed respondents to elaborate on their answers. The IFDH, EDHF, and NVM endorsed the final questionnaire.

### Procedure

The questionnaire was entered in Google Forms, a web-based data entry tool. Only those who received the link via email could open and complete the online questionnaire. The contact details for all national associations were obtained from the IFDH or EDHF websites. Participation was voluntary, and the target group was emailed in November 2018 and informed of the study purpose. The email invitation included the link to the questionnaire and a portable document format (PDF) file of the questionnaire. Therefore, respondents could prepare for the questions before filling out the online form. In addition, information was given regarding the duration of the questionnaire, number of questions, the researchers, and how the data would be used, presented, and published. The participants were also reassured that their contact details would remain anonymous.

A response time of 4 weeks was allowed, and follow-up was conducted through phone calls or a reminder email to prompt associations that did not respond. One last reminder was sent to the nonresponding associations until the questionnaire was closed at the end of December 2018. The responses were automatically saved to a secured database when the respondents completed the questionnaire. Every question required an answer before the next one could be addressed to ensure that the questionnaires were fully completed. The extracted data were automatically entered into a Microsoft Excel spreadsheet. All electronic data were stored safely, and access required a password.

### Data analysis

After obtaining the data, all responding associations were contacted to validate that the research team summarised the data correctly and were asked to provide further clarification of given answers if needed. Data were tabulated and analysed using a descriptive analysis.

## Results

**Fig. 1:**
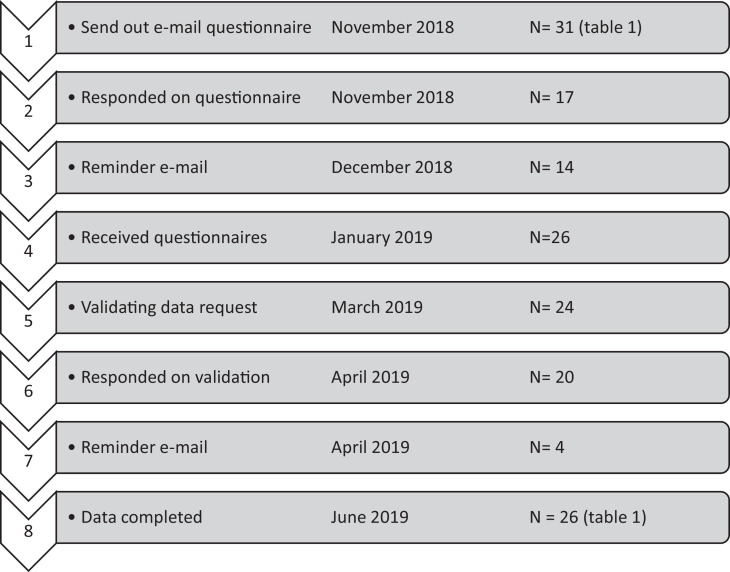
Procedure, time span, and results questionnaire

Thirty-one associations were approached, and 26 responded, resulting in a response rate of 84% (Figure 1). All of the returned questionnaires were completed and considered eligible. Two questionnaires were not answered through Google Forms but were completed in a PDF sent directly by email to the research team. No response was retrieved from the DH associations in Germany, Korea, Nepal, New Zealand, and the United Arab Emirates. Data from multijurisdictional countries are summarised and presented as the common minimum tasks allowed in most states and provinces and marked with an asterisk.

### Organisation of care

The first questions were generic to outline the scope of the professional dental hygiene practice by country. In all but one of the countries, the DH is an officially recognised dental care professional. Only Austria does not recognise dental hygiene as a profession, but DHs in Austria are nevertheless members of the IFDH and EDHF. In 58% of the 26 countries, the DH can work independently, without supervision by a dentist. Direct access for a patient to visit a DH is allowed in 54% of the countries. The highest level of dental hygiene education for the majority of the countries is a diploma or bachelor's degree (Table 2).

**Table 2:** Overview by country of education level, recognition, independent practice, and direct access for the DH profession.

Country	Education Level	Recognised	Independent Practice	Direct Access^1^
Australia*	Bachelor Degree, Other	Yes	No	Yes
Austria	Other	No	No	No
Canada*	Diploma, Bachelor Degree	Yes	Yes	Yes
Czech Republic	Bachelor Degree, Diploma	Yes	Yes	No
Denmark	Bachelor Degree	Yes	Yes	Yes
Finland	Bachelor Degree	Yes	Yes	Yes
Ireland	Diploma	Yes	No	No
Israel	Diploma	Yes	No	No
Italy	Bachelor Degree	Yes	Yes	No
Japan	Associate/Bachelor Degree, Diploma	Yes	No	No
Latvia	Diploma	Yes	Yes	Yes
Lithuania	Associate/Bachelor Degree	Yes	Yes	Yes
Malta	Bachelor Degree	Yes	No	No
Netherlands	Bachelor Degree	Yes	Yes	Yes
Norway	Bachelor Degree	Yes	Yes	Yes
Poland	Diploma/ Associate Degree	Yes	No	No
Portugal	Bachelor Degree	Yes	No	Yes
Russia	Diploma	Yes	No	No
Singapore	Diploma	Yes	Yes	No
Slovakia	Bachelor Degree	Yes	Yes	No
South Africa	Bachelor Degree	Yes	Yes	Yes
Spain	Other	Yes	No	No
Sweden	Bachelor Degree	Yes	Yes	Yes
Switzerland*	Diploma	Yes	Yes	Yes
United Kingdom*	Diploma/ Bachelor Degree	Yes	Yes	Yes
United States*	Associate Degree	Yes	No	Yes
*Percentage*		***96%***	***58%***	***54%***

DH, dental hygienist.

Data from multijurisdictional countries are summarised and presented as the common minimum tasks allowed in most states and provinces and marked with an asterisk.

### Local anaesthesia

In 62% of the countries, the DH can administer local anaesthesia via infiltration and block anaesthesia. More countries allow infiltration anaesthesia (57%) in comparison to block anaesthesia (27%). In a few countries (12%), local anaesthesia by injection is not allowed, but the DH can use topical anaesthesia.

In 35% of the countries, the DH can purchase local anaesthesia, and in 42% the DH can store local anaesthesia. The need for a referral or prescription to administer local anaesthesia provided by a dentist is needed in 27% of the countries. Of the countries that allow independent practice and direct access to the DH, only 6 countries allow the DH to buy, store, and administer local anaesthesia in the form of infiltration. These countries are Canada, Denmark, Finland, Lithuania, Norway, and Sweden.

### Caries treatment

Concerning the scope of practice of the DH in cariology, 23% reported that a DH can indicate, decide on the need for a treatment, the placement of a caries restoration. A smaller percentage (15%) of the countries allow the DH to actually place a caries restoration. The majority of the countries (81%) allow the DH to apply sealants. Of the countries that allow independent practice and direct access (42%) to the DH, 3 countries allow the DH to indicate and place restorations for caries treatment: the Netherlands, South Africa, and Sweden (see Table 4).

### Response to open-ended questions

The countries provided additional information in the open-ended questions to expand on the perspective of their answers. Below are some important responses of multiple countries as an example.

*After a 2-year training, the DH is not allowed to anaesthetise or treat caries in deciduous teeth but with a 3-year training they are allowed to.*

*If you do not have a bachelor degree you have to take additional education in anaesthesia.*

*We expect continuous changes and development since there is no governmental regulation for the dental hygiene profession. It is the education itself that set[s] the limitations of what is allowed and not. There is no formal delegation between regulated professions since you always perform your tasks by taking a personal responsibility. The employer can limit your scope of practice if you lack skills and/or competence.*

## Discussion

This paper summarises the extended scope of practice of the DH regarding the administration of local anaesthesia and treatment of caries in numerous countries worldwide with an organised DH community. Multiple efforts of the research team to limit nonresponse in the targeted group resulted in a response rate of 84%. In the light of a worldwide perspective with almost 200 countries, the number of N = 26 responding countries can be considered limited. However, not every country has legalised the DH profession.^14^ The main influencing factor appears to be the local health care organisation and available workforce in oral health. For instance, the number of dentists in the country may not justify an additional oral health care worker.^18^^,^^19^ Therefore, from a traditional viewpoint, the general DH tasks are performed by dentists.^14^

The IFDH unites DH associations around the world (Table 3). Membership indicates an association's perceived legitimacy and potential influence.^14^ Countries with large populations in Asia, South America, and Africa are not represented in the current data because they were not affiliated with the IFDH and therefore did not meet the inclusion criteria.^11^ An alternative approach for a target group is reviewing all countries globally via the internet for DH associations not affiliated with the IFDH. Nonetheless, these countries’ legal positions and education levels are not easily traceable.

**Table 3:** Overview by country regarding the DH profession and local anaesthesia.

Country	Purchase anaesthetic materials	Own anaesthetic materials	Administer local anaesthesia	Referral	Type of anaesthesia
Australia*	No	No	Yes	No	I-B
Austria	No	No	No	-	None
Canada*	Yes	Yes	Yes	?	I-B
Czech Republic	No	No	No	-	T
Denmark	Yes	Yes	Yes	No	I
Finland	Yes	Yes	Yes	Yes	I
Ireland	No	No	Yes	Yes	I-B
Israel	No	No	No	-	None
Italy	No	No	No	-	T
Japan	No	No	No	-	None
Latvia	Yes	Yes	No	-	T
Lithuania	Yes	Yes	Yes	No	I
Malta	No	No	Yes	No	I
Netherlands	No	Yes	Yes	Yes	I-B
Norway	Yes	Yes	Yes	No	I
Poland	No	Yes	Yes	Yes	I
Portugal	No	No	No	-	None
Russia	No	No	No	-	None
Singapore	No	No	Yes	Yes	I
Slovakia	Yes	Yes	No	No	None
South Africa	No	No	Yes	?	I-B
Spain	No	No	No	-	None
Sweden	Yes	Yes	Yes	No	I-B
Switzerland*	No	No	Yes	Yes	I
United Kingdom*	No	No	Yes	Yes	I-B
United States*	Yes	Yes	Yes	No	I-B
*Percentage*	***35%***	***42%***	***62%***	***27%***	***I: 54% B: 27% T: 12%***

DH, dental hygienist; I, infiltration anaesthesia; B, block anaesthesia; G, general (topical) anaesthesia; ?, unknown.

Data from multijurisdictional countries are summarised and presented as the common minimum tasks allowed in most states and provinces and marked with an asterisk.

**Table 4:** Overview by country regarding the DH profession and cariology.

Country	Indicate restorations	Apply restorations	Type of restoration	Apply Sealants
Australia*	Yes	No	None	Yes
Austria	No	No	None	No
Canada*	No	Yes	P-S	Yes
Czech Republic	No	No	None	Yes
Denmark	No	No	None	Yes
Finland	No	No	None	Yes
Ireland	No	No	None	No
Israel	No	No	None	Yes
Italy	No	No	None	Yes
Japan	No	No	None	No
Latvia	No	No	None	Yes
Lithuania	No	No	None	Yes
Malta	No	No	None	Yes
Netherlands	Yes	Yes	P	Yes
Norway	No	No	None	Yes
Poland	No	No	None	No
Portugal	Yes	No	None	Yes
Russia	No	No	None	Yes
Singapore	No	No	None	No
Slovakia	No	No	None	Yes
South Africa	Yes	Yes	P-C	Yes
Spain	No	No	None	Yes
Sweden	Yes	Yes	P-S-C	Yes
Switzerland*	Yes	No	None	Yes
United Kingdom*	No	No	None	Yes
United States*	No	No	None	Yes
*Percentage*	***23%***	***15%***	***P: 15% S: 8% C: 8%***	***81%***

C, cervical; DH, dental hygienist; P,_primary; S, secondary.

Data from multijurisdictional countries are summarised and presented as the common minimum tasks allowed in most states and provinces and marked with an asterisk.

### Multijurisdictional regions

Several countries are, by constitution, based on states, provinces, or cantons. These regions can have legal regulations that differ, such as in Switzerland, Australia, Canada, the United Kingdom, and the United States.^20^ The study data from multijurisdictional countries are summarised and presented as the common minimum tasks allowed in most states and provinces.

In the United States, the majority of states allow the DH to administer local anaesthetic injections. However, most states require the DH to administer local anaesthesia under the direct supervision of a dentist. This requirement implies that the dentist responsible for the procedure must be present in the clinic and personally diagnose the patient to be treated in person. The dentist must also authorise the procedure and examine the overall health condition after the treatment has been completed before patient dismissal. Only general or indirect supervision is needed in a few states, which means that the dentist does not have to be present in the clinic.^21^ Regional differences in one country also apply, such as in Canada^22^^,^^23^ and Australia.^24^ Hence, the requirements vary by state within one country, and variations between countries are also to be expected.

### Divergence

The International Standard Classification of Occupations (ISCO) does not regard the DH profession as a unique group but includes this within the subgroup “3251-Dental assistants and therapists.”^25^ The EDHF recently stated that the core tasks of the DHs are largely confined to educational and promotional activities relating to preventive oral health and examination and the diagnosis and provision of preventive dental care in a common European framework.^9^ Remarkably, the ISCO regulation listed tasks, such as “preparing cavities” and “placing fillings,” whereas the EDHF has stated that several EU member countries would consider these tasks outside the expected scope of DH practice.^26^ Under the present findings, the EDHF further mentioned that 10 EU states report that the DH may carry out tasks under direct supervision or prescriptions of dentists. In all Nordic countries and other countries, such as Switzerland and the United Kingdom, the DH may carry out certain treatments and tasks either at their own authority or by the prescription of a dentist.^26^

The literature has stated that restorative caries treatment should not be part of the DH focus, partially explaining the lack of research on this topic.^14^^,^^27^ However, this may be different in practice, and the work floor may deviate from this supposition. For example, a study in Denmark reported that, amongst dentist tasks performed by the DH, invasive caries treatment was the most frequently delegated.^28^ Second, a study in Italy reported that 3% of patients stated that they had been treated for caries by a DH, whereas this is considered an unauthorised practice of dentistry and is punishable under the Italian Penal Code.^29^^,^^30^

In Japan, the DH can administer topical anaesthesia. In addition, the DH can provide infiltration anaesthesia for periodontal treatment, but a dentist order is needed. Administering local anaesthesia at their own authority is better regulated within the DH scope of practice than invasive caries treatment under the present findings.^28^^,^^27^

“Independently” is understood to imply that the task is carried out without supervision of a dentist. Objections against this DH independence are focused on patient safety and efficiency. Scarce research appears to substantiate these objections. It appears that the underlying factors against independence relate to financial reasons, opinions about competence level, final responsibility, and other sources from the domain of discussion between DHs and dentists.^5^^,^^4^^,^^7^ A more autonomous scope of practice has a positive and significant association with population oral health.^31^

In contrast to dentistry, dental hygiene is a relatively new profession with a history of just a century. Worldwide, dental hygiene has had multiple educational pathways that lead to registration and consequently entrance into the field of oral care providers by country.^8^^,^^32^ Differences in national legislation can also be considered as components that contribute to the diversity of positioning of the DH profession amongst countries.^33^ A direction for further research is the comparison between statutory constructions and the practical execution of the DH practice.

## Conclusions

This paper provides insight into the extended scope of practice of the DH from a global perspective. Considerable variation exists amongst countries regarding the DH's extended scope of practice. Overall, independently administering local anaesthesia appears to be more accepted as being within the DH scope of practice than are caries removal and the placement of restorations.

## Acknowledgements

The authors express their thanks to the IFDH, EDHF, and NVM for their support of this project. The authors also thank their colleagues in the countries that responded to the questionnaire on behalf of their associations .

## Author contributions

MB contributed to conception and design, acquisition, analysis, and interpretation of the data and drafted the manuscript. EB contributed to conception and design, analysis, and interpretation of the data and critically revised the manuscript. GW contributed to design and analysis and interpretation of the data and critically revised the manuscript. DS contributed to conception and design and acquisition, analysis, and interpretation of the data and critically revised the manuscript. All authors gave final approval and agreed to be accountable for all aspects of the work, ensuring integrity and accuracy.

## Funding

This research received no specific grant from any funding agency in the public, commercial, or nonprofit sectors. The work for this paper was funded by the regular academic appointments of the authors at the Academic Centre for Dentistry Amsterdam (ACTA). ACTA is a joint venture between the Faculty of Dentistry of the University of Amsterdam and the Faculty of Dentistry of the Vrije Universiteit Amsterdam, The Netherlands.

## Conflict of interest

None disclosed.

## Appendix Supplementary materials

Image, application 1
