# Supplementary material for: Anaesthesia and Caries Treatment by Dental Hygienists: A Worldwide Review
Source: Int Dent J. 2022 Oct 17;73(2):288–95. doi: 10.1016/j.identj.2022.08.013 (PMC10023583; doi:10.1016/j.identj.2022.08.013)
Supplement: Supplementary file 1 [file mmc1.pdf]

General Questions

These questions are from a general point of view. They are meant to portrait how the profession of a dental hygienist is set up in your country.

*Vereist

1. **1. From which country are you answering this survey?** *

2. **2. What is your title of employment / role within your health board/federation/association?**

3. **3. Which professions are recognized in your country and are legally allowed to perform oral health care?** *
   Vink alle toepasselijke opties aan.
   - [ ] Dental Specialist (i.e. orthodontist, oral surgeon)
   - [ ] Dentist
   - [ ] Dental Hygienist
   - [ ] Dental Therapist
   - [ ] Profylaxis Assistant
   - [ ] Dental Assistant
   - [ ] Other

4. **4. Are there any differences between a dental hygienist and a dental therapist in your country? If so, please specify the main differences or refer to a document describing the differences.** *

5. **5. What is the current level of education of the dental hygienist?** *
   Vink alle toepasselijke opties aan.
   - [ ] Diploma
   - [ ] Associate Degree
   - [ ] Bachelor Degree
   - [ ] Master Degree
   - [ ] Other

6. **6. What are other education levels of the currently working dental hygienists? (for example, dental hygienists who graduated twenty years ago)** *
Vink alle toepasselijke opties aan.

- [ ] Diploma
- [ ] Associate Degree
- [ ] Bachelor Degree
- [ ] Master Degree
- [ ] Other
- [ ] Not applicable

7. **7. Are dental hygienists allowed to run an independent practice?** *
Markeer slechts één ovaal.

( ) Yes
( ) No

8. **8. Do patients have direct access to a dental hygienist? (meaning, without a referral / assignment from a dentist)** *
Markeer slechts één ovaal.

( ) Yes
( ) No

9. **9. Is the dental hygienist legally allowed to delegate tasks to others while keeping the final responsibility? (for example, delegating oral hygiene instructions to a profylaxis assistant)** *
Markeer slechts één ovaal.

( ) Yes
( ) No

10. **10. Are there any changes in process concerning the scope of practice of dental hygienist or their education within a timeframe of the upcoming 5 years? (especially regarding task redistribution and task delegation)** *

Radiology

The questions in this section are intended to illustrate what tasks dental hygienists are legally permitted to perform regarding oral radiology. If the answer options do not fully apply for the situation in your country, leave your comments underneath the last question.

11. **11. Are dental hygienists allowed to personally own radiographic equipment?** *
Markeer slechts één ovaal.

( ) Yes
( ) No

12. **12. Are dental hygienists allowed to indicate / give justification for radiographs? ***
Markeer slechts één ovaal.

- ◯ Yes
- ◯ No

13. **13. Are dental hygienists allowed to take radiographs? ***
Markeer slechts één ovaal.

- ◯ Yes
- ◯ No

14. **14. Which type of radiographs are dental hygienists allowed to take? ***
Vink alle toepasselijke opties aan.

- ☐ None
- ☐ Bitewings
- ☐ Peri-apical radiographs
- ☐ Panoramic radiograph
- ☐ CBCT-scan

15. **15. Are dental hygienist allowed to formulate a formal diagnosis from radiographs? ***
Vink alle toepasselijke opties aan.

- ☐ No
- ☐ Yes, cariology
- ☐ Yes, periodontology and bone level
- ☐ Other (specify below)

16. **16. Are there any restrictions for radiography (i.e. continued education, working experience, degree, dentists permission etc.) ***

Anesthesia

The questions in this section are intended to illustrate what tasks dental hygienists are legally permitted to perform regarding anesthesia. If the answer options do not fully apply for the situation in your country, leave your comments underneath the last question.

17. **17. Are dental hygienists allowed to purchase anesthetic materials? ***
Markeer slechts één ovaal.

- ◯ Yes
- ◯ No

18. **18. Are dental hygienists allowed to own / store anesthetic materials? ***
Markeer slechts één ovaal.

- Yes
- No

19. **19. Are dental hygienists allowed to administer local anesthesia to their patients? ***
Markeer slechts één ovaal.

- No
- Yes

20. **20. Which type of anesthesia are dental hygienists allowed to administer? ***
Vink alle toepasselijke opties aan.

- [ ] Infiltration
- [ ] Block anesthesia
- [ ] General
- [ ] None
- [ ] Other

21. **21. Are there any restrictions for anesthesia (i.e continued education, working experience, degree, dentists permission etc.)**

Cariology

The questions in this section are intended to illustrate what tasks dental hygienists are legally permitted to perform regarding cariology.

22. **22. Are dental hygienists allowed to indicate restorations (fillings)? (meaning, diagnose and recommend a treatmentplan for caries) ***
Markeer slechts één ovaal.

- Yes
- No

23. **23. Are dental hygienists allowed to apply restorations? ***
Markeer slechts één ovaal.

- Yes
- No

24. **24. Which type of restoration are dental hygienists allowed to apply?** *

Vink alle toepasselijke opties aan.

- [ ] None
- [ ] Primary caries restorations
- [ ] Secondary caries restorations
- [ ] Cervical fillings
- [ ] Occlusal sealings
- [ ] Other (specify below)

25. **25. Are there restrictions with respect to cariology (i.e. continued education, working experience, degree, dental therapist, dentists permission etc.)** *

26. **26. Please leave behind your name and email address for us to contact you in case further clarification on any answers is needed.** *

27. **27. If you have any feedback on this questionnaire, please leave it here. We appreciate your effort!** *

Mogelijk gemaakt door
Google Forms
